# PD-L1/PD-1: new kid on the “immune metabolic” block

**DOI:** 10.18632/oncotarget.20639

**Published:** 2017-09-05

**Authors:** Mirjeta Qorraj, Martin Böttcher, Dimitrios Mougiakakos

**Affiliations:** Dimitrios Mougiakakos: Department of Medicine 5, Hematology and Oncology, University Hospital of Erlangen, Erlangen, Germany

**Keywords:** immune checkpoints, immune metabolism, PD-L1/PD-1

Nowadays, reprogramming of the energy metabolism represents a well-established cancer hallmark. The debate regarding the functional rationale for energetic skewing towards aerobic glycolysis (i.e. the Warburg effect) as observed in various entities is still ongoing. Recent findings suggest the existence of heterocellular metabolic interactions that take place within the tumor microenvironment. In fact, they do not only support tumor growth but are also involved in impairing anti-tumor immunity thus contributing to the so-called tumor immune escape. As of to date two key concepts have been widely studied.

First and as anticipated, cancer cells require high amounts of nutrients such as glucose, glutamine, and tryptophan. At the same time it is well accepted that immune cells possess distinct metabolic characteristics (and requirements) that critically control their function. Immune cells that enter the tumor microenvironment are exposed to a “bioenergetic sink” created by the tumor cells. This competition over substrates leads to nutrient deprivation that negatively impact immune functions (Figure 1A). By way of example, rendering tumor cells more glycolytic has been shown to reduce the T-cells’ efficacy to control tumor growth [1].

**Figure 1:**
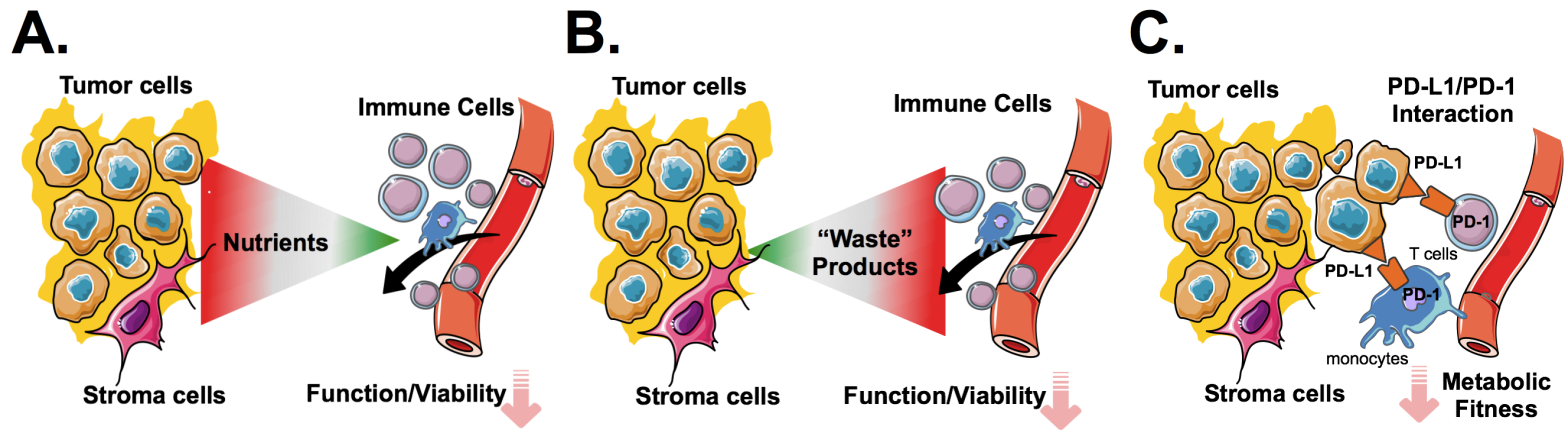
Models of immune metabolic interactions within the tumor microenvironment.

As tumor cells consume and degrade nutrients required for mounting a proper immune response they abundantly release toxic and/or immune regulatory metabolic byproducts that further skew the balance away from anti-tumor activity towards tumor tolerance (Figure 1B). The key mediators of inhibitory metabolic signaling are currently under intense investigation. Enhanced aerobic glycolysis yields lactic acid that negatively impacts T- and NK-cell function [2]. Accumulation of tumor cell-derived reactive oxygen species leads to oxidative stress [3], which is responsible for a wide array of immune dysfunctions.

During the last years we have been focusing on immune metabolic alterations in chronic lymphocytic leukemia (CLL) [3]. Despite the emerging role of monocytes/macrophages as key components for endogenous anti-tumor immunity and as important mediators of the effects triggered by therapeutic antibodies little is known about their immune metabolic fitness in cancer. Similar to activated effector T-cells monocytes/macrophages require a HIF1a- and mTOR-mediated increased glycolytic flux (=glycolytic shift) for functioning efficiently including antibody dependent phagocytosis [4].

We observed that CLL-monocytes display (as compared to healthy control-derived cells) a number of changes in terms of their metabolic phenotype [5]: glucose uptake, glucose transporters at the cell surface, and expression of key glycolytic molecules are significantly reduced. Moreover, CLL-monocytes fail to undergo a glycolytic shift during cytokine-triggered differentiation into type 1 macrophages. For further highlighting the functional importance of glycolysis we pretreated monocytes with glycolytic inhibitors (i.e. 2-deoxy-glucose) or with promoters of glycolysis (i.e. insulin) before performing phagocytosis assays with primary CLL-cells that were opsonized using therapeutic anti-CD20 antibodies. As anticipated, glycolytic rate correlated positively with the monocytes’ efficacy to eliminate the target CLL-cells. Similar findings were made in CLL-derived T-cells that exhibited glycolytic defects with amongst others reduced expression of the glucose transporter 1 and of the critical glycolysis pacemaker enzyme hexokinase 2 [6]. Restoring metabolic competence partially rescued T-cell function and consequently improved anti-leukemic activity.

Interestingly, glycolytic (and phagocytic) defects in monocytes were further aggravated by the Bruton’s tyrosine kinase (BTK) inhibitor ibrutinib that has heralded a new era of targeted therapies in CLL. Those observations support the notion that monoclonal antibodies and current BTK inhibitors might interact negatively when combined. It remains to be seen whether those effects are less pronounced when using the next generation (of more specific) BTK inhibitors.

When analyzing the CLL-monocytes’ immune phenotype we noticed a prominent expression of the immune checkpoint PD-1. Immune checkpoint proteins regulate immune responses for maintaining self-tolerance and for preventing long-lasting or excessive inflammatory reactions. However, cancer cells can disrupt immune responses by (over-)expressing inhibitory molecules such as the ligand for PD-1 (i.e. PD-L1). To date, blocking immune checkpoint molecules ushered in a new era of tumor immune therapies with durable clinical responses. Activating the PD-L1/PD-1 signaling axis was shown to elicit metabolic skewing in T-cells by inhibiting glycolysis [7]. Accordingly, triggering PD-1 on monocytes using bioactive recombinant PD-L1 protein reduced glycolytic rate and hampered their phagocytic activity. Interfering with the PD-L1/PD-1 axis reversed the metabolic and phagocytic dysfunctions of monocytes in presence of PD-L1^pos^ CLL-cells. Our findings could thereby provide one mechanistic explanation for a recently published study that very nicely shows that the PD-1 expression on macrophages correlates negatively with their phagocytic potency against malignant cells, and that blocking the PD-L1/PD-1 cascade in preclinical models increases phagocytosis, limits disease activity, and promotes survival in a macrophage-dependent fashion [8].

Taken together, metabolic deregulation of tumor cells leads to low levels of extracellular metabolites and inhibitory metabolic signaling thereby (indirectly) preventing an adequate immune function. In addition, recent laboratory observations indicate that the interaction of PD-L1 with its cognate receptor PD-1 on T-cells and monocytes inhibits amongst others glycolysis and leads to a condition of so-called immune metabolic anergy (Figure 1C). Restoring immune metabolic competence might represent one of the key modes of actions of immune checkpoints inhibitors. It will be very interesting to investigate whether other immune checkpoint molecules such as Lag-3, VISTA, or TIM-3 exert similar metabolic interferences.

## References

[1] Brand A (2016). Cell Metab.

[2] Chang CH (2015). Cell.

[3] Jitschin R (2014). Blood.

[4] Cheng SC (2014). Science.

[5] Qorraj M (2017). Leukemia.

[6] Siska PJ (2016). J Immunol.

[7] Patsoukis N (2015). Nat Commun.

[8] Gordon SR (2017). Nature.

